# *Salacia* Extract Improves Postprandial Glucose and Insulin Response: A Randomized Double-Blind, Placebo Controlled, Crossover Study in Healthy Volunteers

**DOI:** 10.1155/2016/7971831

**Published:** 2016-10-10

**Authors:** Shankaranarayanan Jeykodi, Jayant Deshpande, Vijaya Juturu

**Affiliations:** ^1^Research and Development, OmniActive Health Technologies Ltd., Mumbai, India; ^2^Research and Development, OmniActive Health Technologies Inc., Prince Edward Island, Canada; ^3^Scientific and Clinical Affairs, OmniActive Health Technologies Inc., Morristown, NJ, USA

## Abstract

Thirty-five healthy subjects were randomly assigned to different doses of* Salacia chinensis* extract (200 mg, 300 mg, and 500 mg SCE) capsules and compared with placebo. It is a placebo controlled randomized crossover design study. Subjects were given oral sucrose solution along with capsules and plasma glucose and insulin responses were analyzed. Blood samples were collected at 0, 30, 60, 90, 120, and 180 minutes after administration. AUC insulin significantly lowered after ingestion of SCE. No significant adverse events were observed. Reducing glucose and insulin is very important in reducing postprandial hyperglycemia.

## 1. Introduction

Obesity is an epidemic in every country. In the U.S., rates of obesity are observed over 20 percent in every state and exceed 25–35 percent in 45 states. In addition, one-in-three adults could have diabetes by 2050. Medical care and cost may increase from $147 billion to nearly $210 billion per year in U.S. [1]. The obesity epidemic long-associated with the Western world is now extending to Eastern nations like China and India. According to the Overseas Development Institute (ODI) about 1.46 billion Asian Indian people are now considered overweight or obese, with a national rate of about 11 percent. India is the third most obese country followed by China [2]. There are several approaches to manage weight by limiting the intake of food and absorption of food, suppressing appetite, and altering metabolism or increasing energy expenditure through physical activity and diet. Dietary supplements are also used for weight management and healthy metabolism and to support healthy lifestyles. Over a decade of basic and clinical research and safety data are available for dietary supplements, including herbal supplements, used for weight management and healthy metabolism.

**Figure 1:**
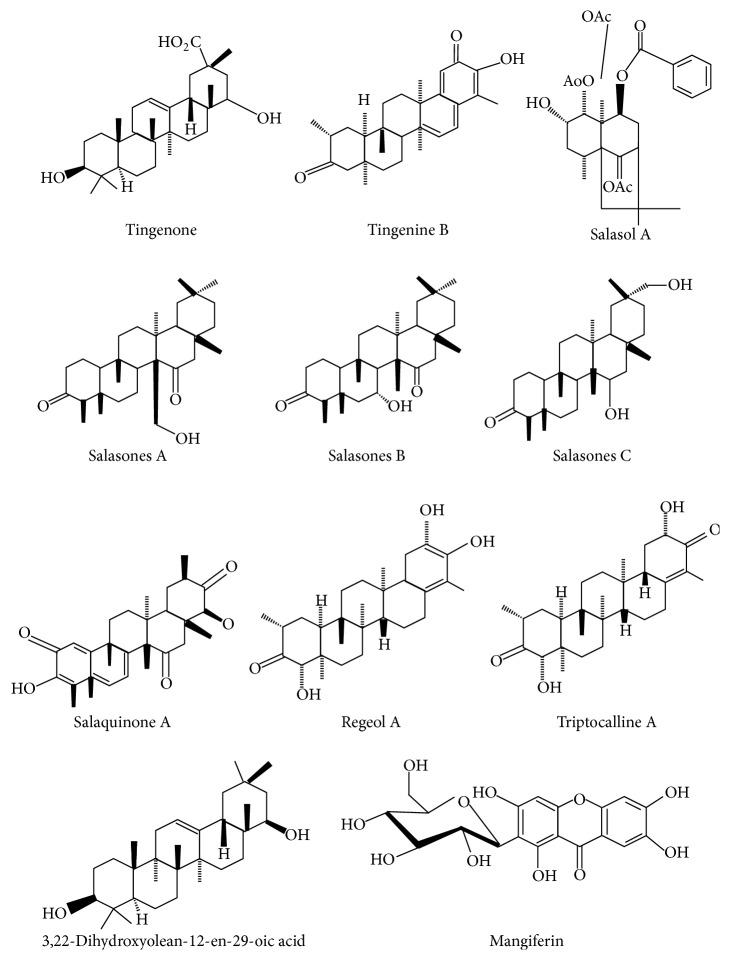
Chemical constituents of* Salacia chinensis* (SCE) [44].

*Salacia chinensis* Linn. (synonyms:* Salacia prinoides*) belongs to Celastraceae (spike-thorn) family commonly called Saptrangi, Dimal, Modhupal, Ingli, Cherukuranti, and Nisul-bondi. It is available throughout India, including Andaman and Nicobar Islands [3]. The roots have biologically active compounds, such as triterpenes, phenolic compounds, glycosides, and coloring agents (Figure 1) which show various medicinal properties.* Salacia* appears to have a fairly unique polyphenolic profile.* Salacia* tends to contain Salacinol, Salaretin, Mangiferin, kotalanol, triterpenes, 13 MRT, and ponkoranol [4, 5].

Recent preclinical and clinical studies have demonstrated that* Salacia* roots modulate multiple targets: peroxisome proliferator activated receptor-alpha-mediated lipogenic gene transcription [6, 7], angiotensin II/angiotensin II type 1 receptor [8], alpha-glucosidase, aldose reductase, and pancreatic lipase [9]. These multitarget actions may mainly contribute to* Salacia* root-induced improvement of type 2 diabetes and obesity-associated hyperglycemia, dyslipidemia, and related cardiovascular complications seen in humans and rodents.

The primary objective of this study was to compare the effect of 3 different doses of* Salacia chinensis* extract (SCE) on the glycemic and insulinemic response in normal healthy individuals. Most of the studies were conducted with healthy and type 2 diabetes with other* Salacia* extract species at higher doses.

## 2. Materials and Methods

### 2.1. Subjects

**Figure 2:**
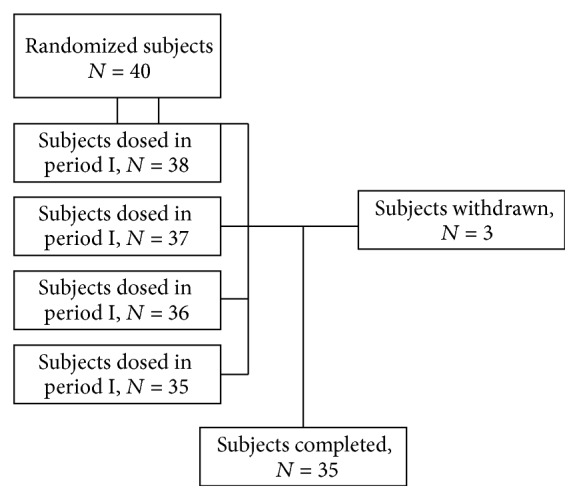
Study design.

Forty eligible subjects (*N* = 40) were healthy South Asian Indian volunteers, consenting adult human male and female subjects, aged 18–55 (both ages inclusive), of BMI 24.5 to 29.5 Kg/m^2^. Subjects were randomly assigned to and completed different treatments (Figure 2). A full medical examination was performed on all subjects, including a physical examination, biochemical tests (routine blood and urine chemistry), and electrocardiogram. An oral glucose tolerance test was also performed in order to confirm normal oral glucose tolerance of each subject. The predose sample (1 × 4 mL) or placebo was administered within 1 hour SCE administration to all the participants. The postdose blood samples (1 × 4 mL) were drawn at 30 min, 60 min, 90 min, 120 min, and 180 min in each period. Participants were required to avoid all dietary supplements, OTC, or any foods that may interfere with postprandial glucose and insulin before and throughout the study period and were not allowed to drink alcoholic beverages or caffeine during the study period. Diet and exercise were strictly controlled. An overnight fast for at least 12 h was required before each treatment.

### 2.2. Study Procedures

Demographic data, medical history, physical and systemic examination, and vital parameters including respiratory rate, EKG, chest X-ray, hematology, biochemistry, serology, and urine analysis were collected or conducted. In addition, serum pregnancy tests were conducted for female subjects.

One capsule of 200 mg SCE (R, T_1_) or one capsule of 300 mg SCE (M, T_2_) or 500 mg SCE (B, T_3_) or placebo (H, T_4_) were given orally to each subject with about 75 g of sucrose in about 250 mL of water at ambient temperature, in each study period, as per the randomization code list. Dosing activity was followed by mouth check to assess the compliance to dosing.

A washout period of 7 days was maintained between dosing days of each periods.

### 2.3. Origin of Raw Material of* Salacia chinensis*

The roots of* Salacia chinensis* are procured from Visakhapatnam. Voucher specimen collected from the source populations is identified and authenticated by in-house botanist at OmniActive. The herbarium sheets are maintained at R & D, OmniActive Health Technologies Ltd., India.

### 2.4. Preparation Method of Extract

100 g of powdered material of* Salacia* roots was extracted with 6 volumes of ethanol at 50 degrees. The ethanolic extract was filtered through Büchner funnel and evaporated to dryness using Rotary evaporator to get yield of 5%. The extract was then analyzed for total polyphenols and Mangiferin content.

### 2.5. Chemical Constituents

Friedelane triterpenes, Friedel-1-en-3-one, Friedelan-1,3, dione 7*α*-ol, Friedelan-1,3-dione- 24-al, Friedelan-1,3-dione-24-oic acid, 24,25-oxidofriedelan-1,3-dione, 7,24-oxidofriedelan-1,3-dione, and 25,26-oxidofriedelan-1,3-dione are isolated from root bark. Mangiferin, Salacinol, kotalanol, Salaprinol, ponkoranol, and leucopelargonidin monomer, its dimer, and tetramer are also reported from roots of* Salacia chinensis* [10].

Based on the totality of the evidence, on the basis of scientific procedures [11], history of exposure, and use, the consumption of* Salacia chinensis *extract (SCE) as a food ingredient at use levels of 50 mg/serving in certain specified foods resulting in a 90th percentile intake of 511 mg/person/day is considered safe and Generally Recognized As Safe (GRAS) [12].

### 2.6. Blood Samples

Blood samples were drawn for glucose and insulin measurements at a central laboratory at the following times: baseline (before product administration) and 30, 45, 60, 90, 120, 150, and 180 min. The serum samples were allowed to clot in serum separator tubes at room temperature and centrifuged at 1000 ×g for 15 min at room temperature. Glucose was measured with the use of an enzymatic method (hexokinase glucose) and insulin was measured with the use of a radioimmunoassay procedure.

### 2.7. *Salacia* Formulation and Dose

One capsule of* Salacia* extract (*Salacia chinensis*, SCE), 200 mg capsules (R, T_1_), or one capsule of* Salacia* extract (*Salacia chinensis,* SCE), 300 mg capsules (M, T_2_), or one capsule of* Salacia* extract (*Salacia chinensis,* SCE), 500 mg capsules (B, T_3_), or one capsule of placebo (H, T_4_) was administered orally to each subject in sitting posture, with about 75 g of sucrose in about 250 mL at ambient temperature, in each study period, as per the randomization code list.

The study protocol was reviewed and approved by the Sri Venkateshwara Hospital Ethics Committee on 07 June 2015, and all enrolled subjects provided informed consent before the start of the study. The study was conducted in compliance with final protocol, the applicable Harmonized Tripartite Guidelines for Good Clinical Practice (GCP), the relevant sections of Good Laboratory Practice (GLP), local laws and regulations (ICMR Guidelines on Biomedical Research), Schedule Y (amended version, 2013) of CDSCO (Central Drugs Standard Control Organization), relevant sections of Drugs and Cosmetics (First Amendment) Rules 2013, CDSCO Bioavailability Bioequivalence Guidance, and the provisions of Declaration of Helsinki (Brazil, October 2013). The trial was registered at ISRCTN # 84979645.

### 2.8. Statistical Analysis

The number of subjects needed to detect difference in the acute study with 80% power at the 5% level of significance was 35–40 subjects for a crossover design. Intention-to-treat (ITT) analysis was conducted. Each variable was analyzed by using parametric or nonparametric (if declared nonnormal) period, treatment, and crossover analysis. The parametric analysis was performed by using repeated measures analysis of variance with variance components covariance structure including treatment and period as fixed effects and subject nested within site as random effect. The pairwise differences of least squares means of the treatments were tested with the use of Tukey-Kramer *p* value adjustments. A result was declared statistically significant if and only if a *p* value of an analysis < 0.05. Statistical software SAS release 8.2 (SAS Institute Inc., Cary, NC) was used for the analyses.

## 3. Results

### 3.1. Baseline Characteristics

A total of 40 subjects were enrolled and participated in the study, out of which 35 subjects completed four periods of the study. All enrolled subjects were healthy human adult male subjects of South Asian race (Indian). Of the 40 subjects enrolled, 38 subjects participated in period one, 37 subjects in period two, 36 subjects in period three, and 35 subjects in period four of the study. A total of 35 subjects completed the study (Figure 2 and Table 1).

### 3.2. Serum Insulin

**Figure 3:**
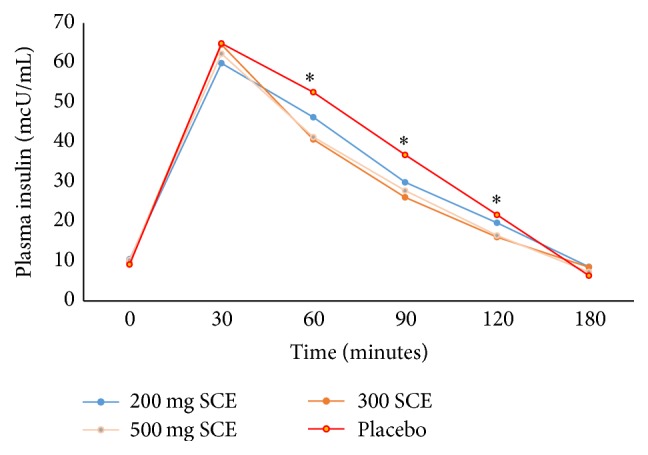
Insulin ↓ with SCE 200 mg followed by SCE 500 mg immediately after sucrose loading at 30 minutes based on ITT analysis. *∗* refers to significance.

No significant differences were observed between treatments for baseline serum insulin concentrations. Baseline and postprandial values for serum insulin are seen in Figure 3 for all available subjects. The mean changes in serum insulin concentrations are shown in Table 2. In addition, two doses of SCE (300 mg and 500 mg) lowered serum insulin area under the curve (AUC) for 0–180 min postprandially in comparison with placebo (Table 4).

### 3.3. Serum Glucose

**Figure 4:**
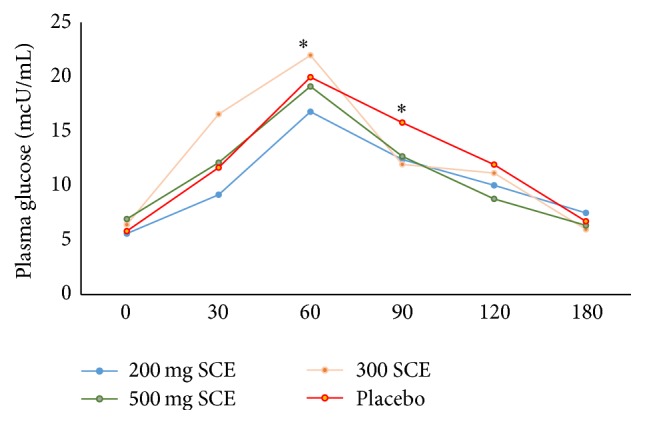
Glucose ↓ with SCE 200 mg (SCE) followed by SCE 500 mg immediately after sucrose loading at 30 minutes. *∗* refers to significance.

No significant differences were observed between treatments for baseline serum glucose concentrations. Baseline and postprandial values for both serum glucose and insulin are seen in Figure 4 for all available subjects. The mean change in serum glucose concentrations is shown in Table 3. In addition, a nonsignificant decrease of serum glucose area under the curve (AUC) for 0–180 min postprandial levels was observed in 200 mg SCE in comparison with placebo (Table 4).

### 3.4. Safety and Gastrointestinal Tract Tolerance

Fever, chills, headache, decreased hemoglobin, decreased hematocrit, increased serum sodium, increased platelet count, and decreased lymphocytes were observed and investigator declared that these are not related to product but they resolved with subjects. No symptoms related to gastrointestinal (GI) tolerance such as flatulence, belching, abdominal pain, nausea, and diarrhea after 24 h and 48 h of product administration were observed.

## 4. Discussion

This study presents the first published results on the effects of SCE on postprandial blood glucose and insulin response in healthy people. The doses of the herbal extract had significant effects on postprandial insulinemia after administration with sucrose solution. The doses of extract for this study are lower than the 1000 mg dose found to be efficacious in other* Salacia* extracts species studies [13–15].

*Salacia *composition described here includes at least 12% of polyphenols, 2% of Mangiferin, and 1% of 25,26-oxidofriedelane-1,3-dione by weight of the composition in the form of extract. The composition is prepared by using nonaqueous food grade solvents. The extract is a proprietary product with specific composition rich in oligomeric flavonoids (proanthocyanidines) [10, 16]. Glycemic status and diabetes complications much correlated in prospective observational studies [17]. Postprandial hyperglycemia is a better predictor of progression to diabetes and key management marker for glycemic control. Avignon et al. [18] reported that postprandial glucose (PPG) based on postlunch plasma glucose and extended postlunch plasma glucose was more reliable in predicting poor glycemic control than prebreakfast or prelunch plasma glucose. The degree of risk conferred by the 2 h PPG concentration was nearly twice that conferred by A1C level [19].

Postprandial insulin levels are diagnostic markers to show insulin resistance and a predictive risk factor for cardiovascular risk. The delayed gastric emptying and a blunted response of gut hormones during feeding may potentially modulate satiety when treated by SCE.

Alpha-glucosidase is an intestinal enzyme which breaks down sucrose into glucose and fructose. Alpha-glucosidase inhibitors class delay and reduce the amount of glucose that is ready for absorption by interfering with the breakdown of the long-chain carbohydrates allowing the pancreas more time to secrete insulin to cover the meal. In the present study, during a sucrose load, SCE reduced insulin in a dose-dependent fashion and glucose was also reduced nonsignificantly over placebo, but not dose-dependently. These results indicate that SCE induces carbohydrate malabsorption. Insulin levels are reduced not only via a decreased glycemic stimulus but also by interference with other insulin releasing mechanism(s).

Therapy with *α*-glucosidase inhibitors can benefit patients with diabetes beyond lowering postprandial glucose. For example, in the STOP-NIDDM (Study to Prevent NIDDM) trial, the group randomly assigned to acarbose not only had a reduction in body weight, BMI, waist and hip circumferences, systolic and diastolic blood pressure, blood triacylglycerols, and 2 h postprandial glucose during a 3 y period following subjects with impaired glucose tolerance but also experienced a significantly reduced incidence of cardiovascular events and hypertension [20]. A meta-analysis of 7 long-term studies showed that *α*-glucosidase inhibitors significantly reduce the risk of myocardial infarction or any cardiovascular event [21].

**Figure 5:**
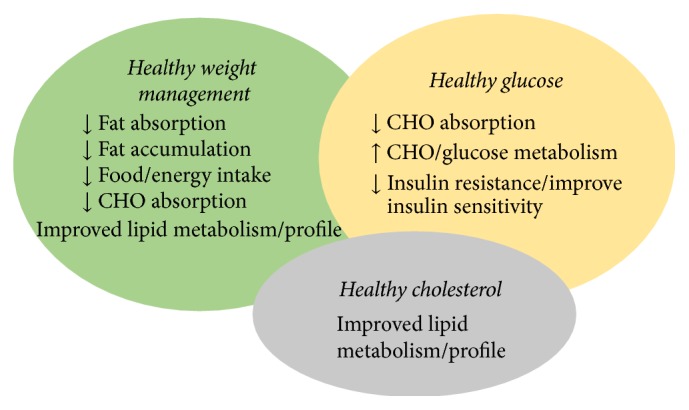
SCE potential mechanism of action on carbohydrate and fat metabolism.

SCE showed significant inhibitory effects on *α*-glucosidase, pancreatic lipase, and HMG-CoA. Potential mechanism of action of SCE observed in the current study might be due to its action as *α*-glucose inhibition (Figure 5). Novel ingredients such as SCE may be ideal for medical nutritional therapy. Lifestyle modifications consisting of diet and exercise can be effective for reducing macrovascular complications in patients with type 2 diabetes and for lowering relative risk of developing the disease in high-risk persons [22, 23]. Although diabetes and its encompassing symptoms are altered by diet and exercise, behavioral obstacles can prevent occurrence of changes. Several situational obstacles for adults with diabetes were identified for dietary adherence, such as resisting temptation, eating out, feeling deprived, planning meals, and social events [24, 25].

In the current study, sucrose loading increased blood glucose concentrations at 30 minutes after administration. Subjects treated with SCE have reduced blood glucose and insulin. Elevation of insulin levels following sucrose loading was also significantly inhibited by SCE compared with placebo; the inhibition was significant at 30 and 60 minutes after administration of 300 and 500 mg SCE. Slowdown of the postprandial hyperglycemic process, if possible, would offer an advantage to insulin-dependent diabetic individuals. Inhibition or delay of intestinal nutrient absorption is now being considered, at least in part, responsible for the hypoglycemic effect. No serious adverse effects were observed after administration of SCE. No risk of lactic acidosis or other serious symptoms commonly seen with hypoglycemic ingredients were observed. SCE does not directly decrease glucose in the blood stream but was shown to inhibit intestinal absorption. Current limitations of the study include the facts that it includes healthy people and administered sucrose solution to study the effects on glucose and insulin postprandial response. They have healthy blood sugar levels and are not abnormal and blood sugar levels came back to normal in all groups at 180 min. Further short-term and long-term studies are required in healthy individuals and metabolic health conditions with and without meal.

Historical and present uses of* Salacia* in India and Japan show that this herbal extract is used as a nutritional adjunct, either as a tea or supplement, taken with meals for its antidiabetic properties.* Salacia reticulata* [23, 26–32] and* Salacia oblongata* [33, 34] do lower postprandial glycaemia in patients with type 2 diabetes and metabolic risk factors [35–37].* Salacia chinensis* with a meal suppressed the increases of postprandial blood glucose at 30 minutes after meal. AUC_glucose_ and AUC_insulin_ in the subjects with a fasting blood glucose level between 100 and 125 mg/dL in healthy Japanese volunteers [38]. In another study [13], 1000 mg extract of* Salacia chinensis *was given with carbohydrate-rich diet (approximately 600 Kcal) and AUC glucose decreased and no data are available on insulin. These two studies were reported with a meal and current study is with a sucrose loading as recommended by FDA for alpha-glucosidase inhibitor products and sucrose (rather than starch) was the most appropriate carbohydrate load [39]. As a disaccharide, sucrose cannot be systemically absorbed unless it is hydrolyzed to glucose and fructose by *α*-glucosidase. After a dose of SCE, the decrease in absorption of glucose produced from sucrose reflects the activity of *α*-glucosidase and indirectly reflects the efficacy of SCE. So administration of sucrose was recommended to provide a better baseline measure of *α*-glucosidase activity. Koytchev et al. [40] administration of sucrose is more suitable than eating a meal since it produces a more reproducible change in serum glucose concentration. Studies on low dose SCE were not reported in healthy people nor in disease condition. In a pilot study, the effects of* S. chinensis *were investigated in diabetic chronic kidney disease (CKD) patients [41]. In this study, 30 stable diabetic CKD patients were randomized into 2 groups: groups A and B of 15 patients each. Group A was given* S. chinensis* 1000 mg twice daily, while group B received a placebo. There was stabilization of renal function as measured by serum creatinine and creatinine clearance in patients receiving* S. chinensis* compared to the placebo, suggesting that* S. chinensis* may retard the progression of chronic kidney disease. Similarly, there was a significant decline in both serum homocysteine and IL-6 levels. In a randomized double-blind, placebo controlled, crossover study, 30 healthy human subjects were given a placebo or 1000 mg of an* S. chinensis* hydroalcoholic extract as a one-time dose [13]. The extract decreased postprandial plasma glucose levels after a carbohydrate-rich meal by about 13% at 90 min, while the plasma glucose area under the curve was decreased by about 34%. In a double-blind, placebo controlled, randomized trial, Shivaprasad et al. [31] evaluated the efficacy and safety of* S. reticulata* leaves and root bark extracts in 29 subjects with prediabetes and mild to moderate hyperlipidemia. In this study, 29 subjects received either 500 mg/day of a* S. reticulata* extract or a placebo along with therapeutic lifestyle changes for the 6-week period. As compared to the placebo, improvements in lipid profiles and glycemic levels were observed in the* S. reticulata*-treated group at week 6. A statistically significant reduction was observed in low-density lipoprotein cholesterol and fasting blood sugar levels at weeks 3 and 6 when treated with root bark extract. The leaves extract-treated group showed statistically significant reduction in fasting blood sugar levels at week 6 only.

In a double-blind study, Ozaki et al. [5] investigated the safety of drinks containing an aqueous extract of* S. reticulata*. In this study, a total of 54 subjects either healthy (*n* = 27) or with borderline blood glucose levels and mild type 2 diabetes (*n* = 27) were randomly assigned to untreated (placebo) or treated groups. The subjects consumed a drink containing the placebo or* S. reticulata* extract (450 mg; 3 times the recommended amount) at breakfast and dinner every day. The treated subjects showed no significant clinical changes or adverse effects, such as hyperglycemia or gastrointestinal symptoms, during the entire test period. The subjects with borderline blood glucose and mild type 2 diabetes in the treated group showed significant changes in the amount of HbA(1c) and glycoalbumin during the test period compared to the placebo group. In another double-blind randomized placebo controlled trial, Singh et al. [41] investigated the effects of a herbal tea containing* S. reticulata* in patients with type 2 diabetes mellitus. In this six-month study, 51 subjects with type 2 diabetes mellitus for longer than 6 months and with evidence of stable glycemic control over the preceding 6 months participated. The subjects were randomized to receive a standard preparation of* S. reticulata* tea for 3 months followed by placebo in similar tea bags for a further 3 months (*n* + 28) or in reverse order (*n* − 23). HbA1C was measured at recruitment, at 3 months, and on completion of the study at 6 months. There were no significant differences between the two groups in age, body mass index, male/female ratio, glycemic control, and baseline laboratory tests. In a placebo controlled, crossover trial, Kajimoto et al. [29] investigated the clinical usefulness of* S. reticulata* extract for prevention or treatment of type 2 diabetes. The study subjects were 20 individuals (10 males and 10 females, average age 58 ± 15.5 years) with type 2 diabetes. The subjects were divided into two groups and were treated with either the extract containing diet (240 mg/day) or a control diet (placebo) for six weeks. The results indicated that the* S. reticulata* extract containing diet achieved significant reductions in fasting plasma glucose levels, in HbA1C, and in BMI. In a randomized, double-masked, crossover design trial, Heacock et al. [42] investigated the effect of different doses of* S. oblonga* extract on postprandial glycemic, insulinemic, and breath hydrogen responses in healthy adults. In this study, 39 nondiabetic subjects participated in four separate 3-hour meal tolerance tests. The volunteers, after fasting for 12 hours, consumed four test meals consisting of 480 mL of study beverage (14 g fat, 82 g carbohydrate, and 20 g protein) with 0, 500, 700, or 1000 mg of* S. oblonga *extract on four separate occasions. The results from this study showed that, compared with the control, the 1000 mg* S. oblonga *extract dose reduced the plasma glucose and serum insulin incremental areas under the curve (0 to 120 minutes postprandial) by 23% and 29%, respectively. The lower doses of* S. oblonga *extract did not affect glycemia or insulinemia. Breath hydrogen excretion increased linearly as the dose of* S. oblonga *extract was advanced. The investigators stated that “this type of ‘dose-related' effect where the higher doses have greater effect tends to bolster the confidence researchers can place in the results of a study.” In a randomized, double-masked, crossover design, Collene et al. [15] evaluated the effects of an* S. oblonga* extract taken at a dose of 1000 mg daily. In this study, 43 healthy people were given a high carbohydrate beverage with or without addition of* S. oblonga*. The results showed that when the* S. oblonga* extract was included, the normal rise in blood sugar and insulin following consumption of the beverage was significantly decreased. As described in an Honors Thesis, Washam [43] investigated the effects of* S. oblonga* extract on the postprandial glycemic and lactate responses along with perceived gastrointestinal, satiety, and flatulence symptom severity following a solid, high starch meal. In this study, 14 nondiabetic individuals (8 males and 6 females) participated. The results of the study showed that* S. oblonga* extract lowered the postprandial glucose response to a higher starch meal (spaghetti noodles, meatless spaghetti sauce, and unsweetened caffeine-free tea; 480 mg of* S. oblonga* extract was added to the tea for the treatment meal). In a randomized, double-blind crossover study with* S. oblonga*, Williams et al. [34] evaluated the effect of a* S. oblonga* extract on postprandial glycemia and insulinemia in 61 patients with type 2 diabetes following ingestion of a high carbohydrate meal. In a fasted state, subjects consumed one of 3 meals: a standard liquid control meal, a control meal + 240 mg* S. oblonga* extract, and a control meal + 480 mg* S. oblonga* extract. The results from the study showed that both doses of the* S. oblonga* extract significantly lowered the postprandial positive area under the glucose curve and the adjusted peak glucose response compared to the control meal with the higher dose performing better. Both doses of the* S. oblonga* extract also lowered the positive area under the insulin curve in comparison with the control meal with the higher dose performing better. So, the long-term benefits of this herbal extract on glycemic control was explored within this population to find its value in the realm of nutritive therapy.

## 5. Conclusion

SCE markedly decreased digestion and absorption of sucrose by its inhibitory action on sucrase and then reduced increases in blood glucose and insulin without serious adverse effect. Therefore, SCE might afford a safe and effective supplementary means for controlling metabolic health and healthy blood glucose and insulin levels.

## Figures and Tables

**Table 1:** Baseline Characteristics.

Description	Mean ± SD (range)
Age	34.08 ± 6.5 years (21 to 44 y)
Height	1.594 ± 0.081 m (1.448 to 1.746 m)
Weight	65.53 ± 8.31 kg (52 to 84.2 kg)
BMI	25.69 ± 1.64 kg/m^2^ (23.16 to 29.36 kg/m^2^)
Nonsmokers	100%
Nonalcoholics	100%
Nonvegetarians	100%

**Table 2:** Mean change in plasma insulin at different time points between the four treatments (*µ*U/mL).

Treatment	0	30	60	90	120	180
SCE 200 mg	4.03 ± 3.5	23.31 ± 18.69	27.32 ± 18.58	18.85 ± 15.60	11.08 ± 9.11	4.33 ± 4.55
SCE 300 mg	3.57 ± 2.8	24.42 ± 23.08	23.86 ± 20.86	16.90 ± 15.41	9.21 ± 9.67	4.23 ± 4.07
SCE 500 mg	3.70 ± 3.1	21.45 ± 20.72	21.84 ± 18.68	18.09 ± 15.91	9.12 ± 9.40	2.52 ± 2.01
Placebo	2.91 ± 2.1	29.59 ± 24.86	30.90 ± 17.03	26.96 ± 16.27	15.67 ± 11.79	2.15 ± 1.47

**Table 3:** ↓ Plasma glucose was observed in 500 mg SCE at 120 min and 300 mg SCE at 180 min over placebo.

Treatment	0	30	60	90	120	180
SCE 200 mg	5.60 ± 3.35	9.17 ± 8.62	16.80 ± 12.30	12.41 ± 10.19	10.04 ± 6.65	7.50 ± 5.56
SCE 300 mg	6.43 ± 5.05	16.57 ± 10.90	21.98 ± 12.90	11.96 ± 9.44	11.16 ± 8.85	5.99 ± 6.02
SCE 500 mg	6.94 ± 4.93	12.12 ± 9.64	19.12 ± 13.50	12.70 ± 10.29	8.79 ± 6.69	6.35 ± 4.56
Placebo	5.83 ± 3.69	11.66 ± 8.71	19.97 ± 15.64	15.80 ± 9.70	11.94 ± 8.44	6.73 ± 4.76

**Table 4:** ↓ ΔAUC of plasma Insulin in all SCE doses and ↓ ΔAUC Glucose in 200 mg SCE versus placebo.

Treatment	AUC_Insulin_ *µ*U/L/180 min	AUC_Glucose_ mmol/L/180 min
200 mg SCE (R )	2481.71 ± 1701.52	1411.74 ± 1051.02
300 mg SCE (M)	2037.04 ± 1724.16^*∗*^	1811.43 ± 1224.66
500 mg SCE (B)	1992.09 ± 1506.98^*∗∗*^	1647.51 ± 1150.06
Placebo (H)	3135.05 ± 1632.36	1530.85 ± 1192.74

B versus H  *p* < 0.0046^*∗∗*^; R (T_1_): 200 mg SCE; M (T_2_): 300 mg SCE; B (T_3_): 500 mg SCE; H (T_4_): Placebo; *∗* refers to significance.
